# Cholecyst-jejunostomy for palliative surgery

**DOI:** 10.1016/j.ijscr.2021.01.020

**Published:** 2021-01-15

**Authors:** Hideki Kogo, Hideaki Takasaki, Yoshinori Sakata, Yoshiharu Nakamura, Hiroshi Yoshida

**Affiliations:** a Department of Gastrointestinal and Hepato-Biliary-Pancreatic Surgery, Nippon Medical School Tama-Nagayama Hospital, Tokyo, Japan; b Department of Surgery, Kamisu Saiseikai Hospital, Ibaraki, Japan; c Department of Gastrointestinal and Hepato-Biliary-Pancreatic Surgery, Nippon Medical School, Tokyo, Japan

**Keywords:** Case report, Cholecyst-jejunostomy, Palliative surgery, Pancreatic cancer, Obstructive jaundice

## Abstract

**Introduction and importance:**

Many cases of unresectable cancer that cause obstructive jaundice require treatment. Depending on the patient's condition in these cases, surgery may be performed to treat jaundice. The main goal of palliative surgery is to improve the quality of life. Therefore, palliative surgery for obstructive jaundice must be performed safely and quickly.

**Case presentation:**

This case presents a 45-year-old man with fever and back pain who was diagnosed with pancreatic head cancer and multiple liver metastases. Chemotherapy was initiated; however, during the course of treatment, the patient developed hemorrhage from pancreatic cancer that had invaded the duodenum caused hematemesis and melena. Therefore, the chemotherapy could not be continued. Because the patient also developed obstructive jaundice and cholangitis, a gastrojejunostomy and cholecyst-jejunostomy was performed. The surgery was successful; however, the cancer continued to progress, and patient died 31 days after surgery.

**Clinical discussion:**

Biliary reconstruction can be difficult to perform safely and quickly due to many factors.

This study shows that cholecyst-jejunostomy is effective for patients with end-stage cancer. In the long term, cholecyst-jejunostomy is not suitable for biliary reconstruction due to the possibility of bile congestion and cholecystitis. However, this easy and quick procedure is well indicated for emergency patients with a short life expectancy.

**Conclusion:**

As an easy and quick procedure for emergency patients with a short life expectancy, jejunal anastomosis of the gallbladder is an appropriate palliative surgery that is indicated for jaundice treatment.

## Highlights

• Many cases of unresectable cancer that cause obstructive jaundice require treatment.
• Biliary reconstruction can be difficult to perform safely and quickly due to many factors.
• Cholecyst-jejunostomy may be completed within 10 min.
• Cholecyst-jejunostomy is an appropriate palliative surgery.

## 1 Introduction

Many cases of unresectable cancer that cause obstructive jaundice require treatment. In some cases, the obstruction is caused by bile duct or pancreatic head cancer, whereas bile duct stenosis may be caused by gastric cancer with hepatoduodenal-mesenteric lymph node metastasis, resulting in obstructive jaundice [1,2]. Depending on the patient's condition, surgery may be performed to treat jaundice or nonsurgical treatment may be chosen, such as drainage through an external fistula or treatment by endoscopic retrograde cholangiopancreatography (ERCP) [[1], [2], [3], [4], [5]]. If surgery is thought to shorten the patient's life expectancy, then palliative treatment without surgery is chosen [2,3,6]. If surgery is to be performed, then palliative surgery is required because performing radical surgery is not possible. Palliative surgery does not significantly extend the patient's life expectancy [7], and therefore must be performed safely and quickly [2,4,5].

In the present study, palliative surgery was performed for obstructive jaundice and cholangitis in a 45-year-old man with unresectable pancreatic head cancer with multiple liver metastases. To reconstruct the biliary tract, a cholecyst-jejunal bypass was chosen as a surgical approach that can be performed safely and quickly. Although cholecyst-jejunal bypass is a rare procedure, it has been widely performed in the past and was an appropriate choice for the patient in the present case [4,5,9]. Cholecyst-jejunostomy may be suitable for palliative surgery.

## 2 Presentation of case

A 45-year-old man presented to the emergency department because of fever and back pain.

The patient had been in his usual state of health until a week before this evaluation, when

subjective fever, and back pain gradually developed.

**Fig. 1:**
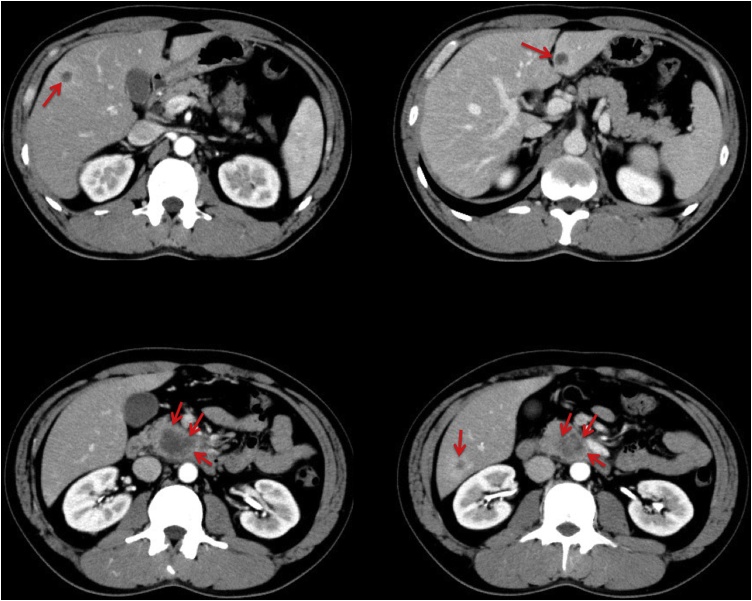
Enhanced computed tomography scan demonstrates pancreatic head cancer and multiple liver metastases.

A plain computed tomography (CT) scan of the abdomen revealed pancreatic tumor and he was referred for surgery. An enhanced CT scan revealed pancreatic head cancer and multiple liver metastases (Fig. 1). The decision was made to initiate chemotherapy (gemcitabine + nab-paclitaxel) for unresectable pancreatic head cancer.

**Fig. 2:**
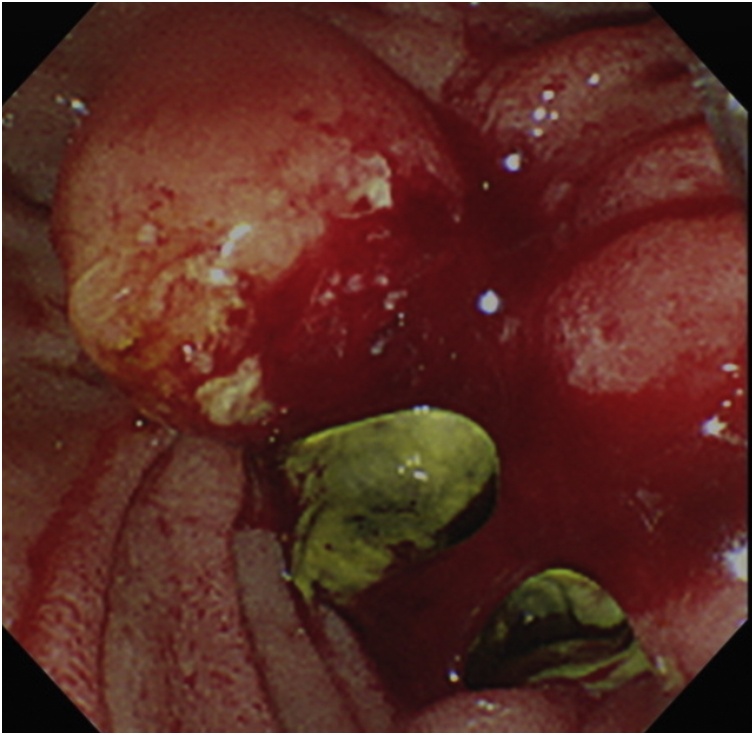
Upper gastrointestinal endoscopy reveals bleeding from pancreatic head cancer that invaded the duodenum.

The patient completed 4 courses of the chemotherapy and was determined to have stable disease. One day, the patient presented to the night outpatient clinic with hematemesis. An emergency upper gastrointestinal endoscopy showed bleeding from the pancreatic head cancer that had invaded the duodenum (Fig. 2). However, because no active bleeding was evident, the endoscopy was finished by spreading thrombin solution to the lesion without hemostatic treatment. After that treatment, the hematemesis stopped, and patient's condition improved by stopping oral intake and administering a blood transfusion.

It was determined that the patient was unable to take anything orally other than water, and the medical team planned to perform gastrojejunostomy on a standby basis. However, 2 days before surgery, the patient left the hospital briefly for during a short outing to return home, where he experienced significant melena. After immediately returning to the hospital, a total of 24 units of packed red blood cells were transfused for treatment of hemorrhagic shock. After discontinuation of oral intake and administration of a proton pump inhibitor, the patient’s general condition recovered; however, the symptoms of liver dysfunction, jaundice, and fever were manifested.

The patient was diagnosed as having developed biliary obstruction due to a pancreatic tumor that caused cholangitis. For the treatment of the jaundice, ERCP was performed. However, deformity of the Vater papillae due to pancreatic tumor invasion was visualized during the procedure, and it was impossible to cannulate via the Vater papillae to continue treatment. Percutaneous gallbladder drainage was then performed and able to reduce the jaundice.

The medical team considered performing palliative care without surgical treatment in this case; however, the patient and his family wanted palliative surgery to be performed and then the patient to be discharged—they spoke of their strong hope for the patient to be able to take oral intake and stay home. To facilitate this care plan, the medical team planned to perform gastrojejunostomy with biliary reconstruction. In this case of advanced pancreatic cancer with concomitant cholangitis, it was presumed that it would not be easy to perform a bile duct–jejunostomy quickly and safely. In addition, the patient's prognosis was considered to be poor, with estimated survival of only about 1 month, so a fast and safe surgical technique was most needed.

**Fig. 3:**
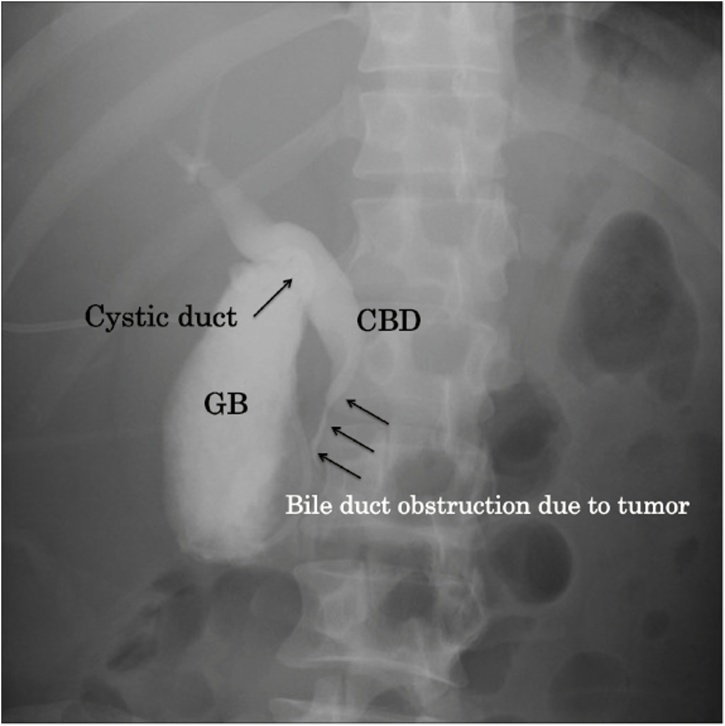
A contrast study via the percutaneous gallbladder drainage tube shows stenosis of the lower bile duct. The cystic duct is well defined.

A preoperative contrast study using the percutaneous gallbladder drainage tube showed stenosis of the lower bile duct. The cystic duct was well defined. No gallbladder stones were found (Fig. 3). Based in these findings, biliary reconstruction with an anastomosis of the gallbladder and jejunum was planned.

### 2.1 Operative findings

The upper median incision was made. Multiple liver metastases on the liver surface were identified. No peritoneal dissemination was found. After opening the bursa cavity, the right gastroepiploic artery and right gastric artery were identified, ligated, and then dissected. The surgical team was able to confirm the gastric pyloric ring.

**Fig. 4:**
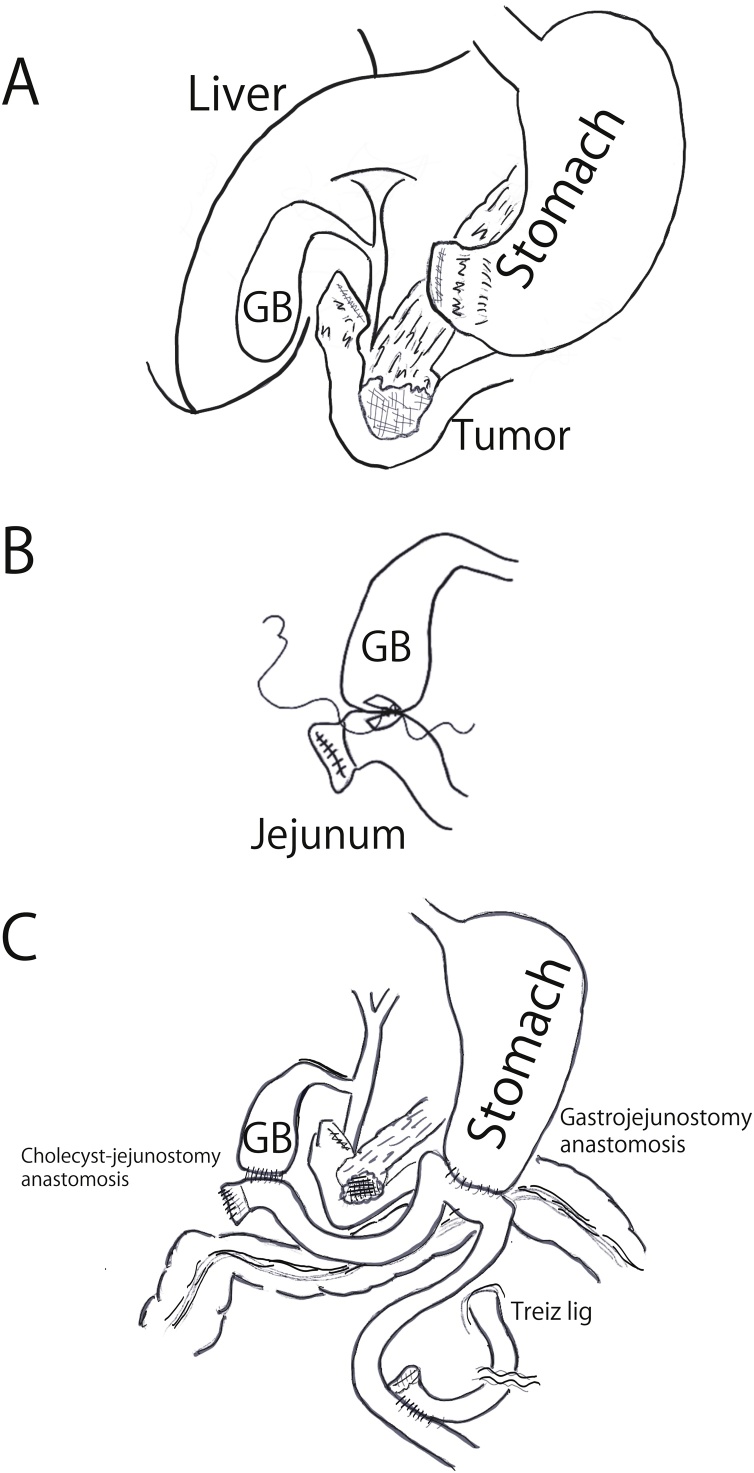
Surgical portrait: (A) The gastric pyloric ring is confirmed. The duodenum side of stomach is dissected using a linear stapler, and the resection margins of the duodenum are embedded with sutures. (B) Cholecyst-jejunostomy with running sutures is performed. (C) A gastrojejunal bypass is performed to create a pathway by which oral intake never passes through the duodenum.

Then, the duodenum side of stomach was dissected using a linear stapler (Fig. 4A). The resection margins of the duodenum were embedded with sutures. The area surrounding the common bile duct was solidly inflamed, which complicated the goal of bile duct–jejunum anastomosis. To shorten the operative time and minimize surgical invasion, the surgical team decided to perform a gallbladder–jejunostomy rather than a bile duct–jejunostomy.

The jejunum was resected 70 cm from the ligament of Treitz and raised in front of the colon. The following next steps were taken: (1) the gallbladder and jejunum were anastomosed using 4-0 polydioxanone running suture (Fig. 4B); (2) gastric and jejunal anastomosis was performed with a linear stapler; and (3) jejunum–jejunum anastomosis was also performed with a linear stapler. To avoid inducing duodenum tumor hemorrhage, a gastrojejunal bypass was created to serve as a pathway in which oral intake never passes through the duodenum (Fig. 4C). The operative time was 134 min, and the blood loss was minimal.

### 2.2 Postoperative course

**Fig. 5:**
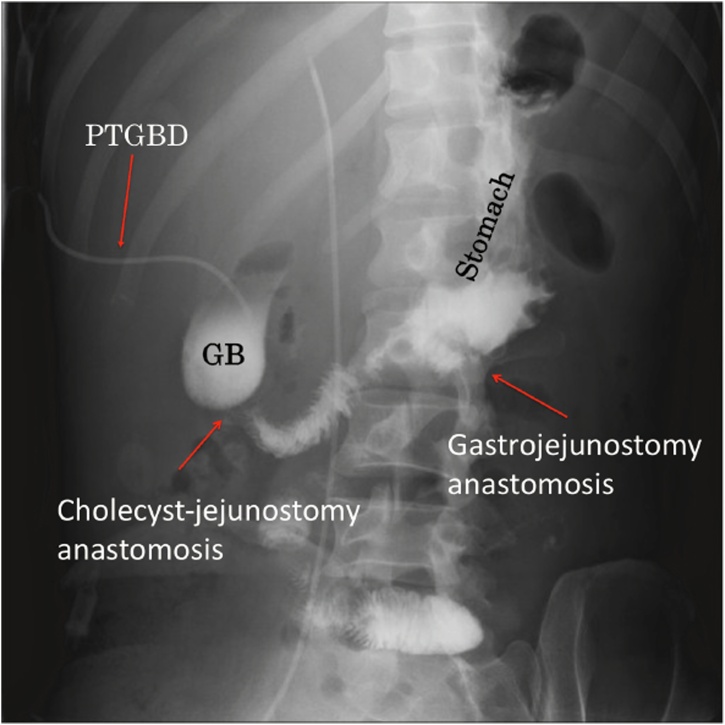
A contrast study via the percutaneous gallbladder drainage tube and upper intestinal contrast study are performed on postoperative Day 11.

The patient was able to get out of bed and walk in the early postoperative period. Drinking water was also started on postoperative Day 2. On postoperative Day 9, the patient was able to be discharged home, and oral intake was started the next day. Follow-up CT on postoperative Day 10 showed an increase in the number of liver metastases in a short period of time. A contrast study by the percutaneous gallbladder drainage tube and an upper intestinal contrast study were performed on postoperative Day 11. The results showed good bile flow and no leakage (Fig. 5). The patient remained home through postoperative Day 18, but then returned to the hospital because of extreme fatigue. By that time, his liver dysfunction had worsened, and his amount of food intake had gradually decreased. On postoperative Day 25, the patient’s route of nutrient administration was switched to completely intravenous. The following day, a do-not-resuscitate order was obtained for the family. The patient then died on postoperative Day 31. The case has been reported in line with the SCARE criteria [8].

## 3 Discussion

Cancer emergencies in terminally ill patients are incurable and have a poor prognosis. In particular, surgery for such patients does not significantly increase life expectancy and may even shorten it [2,5,6]. However, in many of these cases, the patients and their family are often perplexed by the sudden deterioration in the patient’s condition and wish to receive as much treatment as possible, even if only for a short time.

This case presented an otherwise healthy 45-year-old man undergoing treatment for terminal pancreatic cancer who developed tumor bleeding and jaundice and had to be treated as an emergency. The medical team was asked to make a quick decision on how to respond to sudden gastrointestinal bleeding, jaundice, and cholangitis in a patient with end-stage pancreatic cancer. Gastrointestinal bypass surgery is a relatively simple procedure that can be performed in many patients; however, biliary reconstruction can be difficult because of factors such as cancer invasion, inflammation, and adhesions [5].

This study showed that cholecyst-jejunostomy is effective in patients with lower bile duct obstruction [4,5,9]. The surgery is performed with running sutures and may be completed within 10 min (Fig. 4B). Some reports have described cholecyst-jejunostomy performed laparoscopically or with a linear stabler [4,5]. In the long term, cholecyst-jejunostomy is not suitable for biliary reconstruction because of the possibility of bile congestion and cholecystitis [9]. For emergency patients with a short life expectancy, however, it is an easy and quick procedure to perform as palliative surgery [4,9].

## 4 Conclusion

For emergency patients with a short life expectancy, jejunal anastomosis of the gallbladder is an easy and quick procedure to perform as palliative surgery, including for the indication of jaundice treatment.

## Declaration of Competing Interest

The authors declare that they have no competing interests.

## Funding

None.

## Ethical approval

Not applicable.

## Consent

Informed consent was obtained from the patient for the publication of this case report and accompanying images.

## Author contribution

HK: Drafted the manuscript.

HK, YS, and YN: performed the operation.

HK, YS, HT, YN, and HY: Revised the manuscript.

HK, YS, HT, YN, and HY: Read and approved the final manuscript.

## Registration of research studies

Not Applicable.

## Guarantor

On the behalf of all author I am the guarantor. Hideki Kogo.

## Provenance and peer review

Not commissioned, externally peer-reviewed.
